# Impact of traditional healers in the provision of mental health services in Nigeria

**DOI:** 10.1016/j.amsu.2022.104755

**Published:** 2022-09-24

**Authors:** Omolayo Anjorin, Yusuf Hassan Wada

**Affiliations:** Institute of Psychiatry, Psychology & Neuroscience, Kings College London, United Kingdom; Faculty of Pharmaceutical Sciences, Usmanu Danfodiyo University, Sokoto, Nigeria; West African Institute of Public Health, Abuja, Nigeria

**Keywords:** Traditional healers, Mental health, Nigeria, Mental illness, Health systems

## Abstract

Mental health remains one of the most overlooked issues in Nigeria. The burden posed by traditional healers in the provision of mental health services is becoming a public health concern in the country. This article presents a review of the present situation on the mental health service provision in Nigeria by traditional healers and highlights way forward actions for policy makers, government, and other stakeholders. These include the need for a policy direction on the need for establishing an improved system of psychiatric and mental health services in hospitals that will drive cultural acceptability, accessibility, and affordability. Further, there is a need for policy measures to be in place towards creating long term directions and sustainability of documentations of traditional healer's activities, harmful avoidance and improved mental health services in primary health centers. The fact that traditional healers are indispensable in provision of mental health services, call for collaborative effort to improve services such as behavioral change, capacity building and referral practice that could save more lives and reduce severity of complications.

## 1 Introduction

In many parts of the world, particularly Africa, mental health issues are overlooked, misunderstood, mismanaged with a dearth of research into mental health problems. The etiology of mental illness is frequently attributed to culturally acceptable beliefs about divinity, witchcraft, sickness, ancestral spirits' influence, and social wrongdoing [1]. As a result, many people with mental illnesses prefer to seek the services of African traditional healers on the road to receiving mental health services or as their primary source of mental health care [2]. The pathways to mental health care seeking in African countries are influenced by factors at multiple levels, including the individual, societal, organizational, and health system levels [3].

In Africa, traditional healers far outnumber medical doctors, with one traditional healer per 500 population compared with one medical doctor per 40,000 population [4]. This makes traditional healers more available, affordable, and flexible, in comparison with formal mental health workers, facilities and prescriptions. Individuals are also hesitant to obtain professional mental health care due to fear of being diagnosed with mental illness, skepticism of the formal mental health system, lack of faith in health professionals and their personal belief [5]. Traditional healers constitute a significant source of care, they provide a belief system that is complementary to individuals’ cultural belief, in terms of the causes of mental health problems and, as a result, the right therapy for them.

Therefore, traditional healers will continue to have a substantial role in mental health-care delivery because they are not only available and accessible in the community, but they also constitute part of the community's cultural belief system, making them a vital part of the community. Thus, it is important to explore the role of traditional healers on the provision of mental health services and provide recommendations for better practice, particularly in Africa's most populous country, Nigeria.

## 2 Traditional healers in the provision of mental health services in Nigeria

In Nigeria, approximately 80% of people with serious mental health illness are unable to access mental health care and these services are even more limited in rural and underserved areas [6]. Further, this is even challenging with a workforce of fewer than 300 psychiatrists in a country of over 200 million people and a diversity of more than 250 dissimilar cultural ethnic groups, with each having its indigenous ways of ensuring health to its people [7].

The etiology and treatment of mental illness is understood in a variety of ways (including possession by spirits), people may migrate between health centers and traditional healers. Witchcraft and ancestor spirits are common cultural beliefs thought to have caused mental health illness and this guides their health seeking behaviors [8]. Families of people with mental health disease are left to choose between mental health practitioners and healers. As a result of the insufficient community-based formal mental health treatments, faith-based (or prayer camps) and traditional healers have emerged as a comparatively desirable and practical alternative for mental health therapy [9]. The popularity of selecting traditional healers is therefore compounded by factors such as limited accessibility, availability, and affordability of mental health in the overall country's healthcare system, and stigma and poor understanding of the disease [10].

Agara et al. [11] investigated the understanding of mental disease in Nigeria and discovered that, among other things, mental illness was linked to witchcraft (93.3%), punishment for sins (73.3%), and supernatural causes (66.7%). This misconception of mental health disorders often prompt relatives to take their loved ones to religious or traditional healing place. As a result, individuals who develop mental problems receive treatment from traditional leaders, churches, prayer camps, and imams around Nigeria [12]. In a study of about a 1000 Nigerians, for example, approximately 80% of people with mental problems sought mental health care from informal providers such as priests, spiritualists, or traditional healers [13]. Furthermore, nearly 70% of patients diagnosed with schizophrenia in Lagos, Nigeria, are documented to have sought therapy for their mental disorders through spiritualists or traditional healers [14].

The traditional remedies for treating mental illness include both pharmacological and non-pharmacological approaches which includes herbs, spiritual therapy, counseling, and psychotherapy [15]. In a study in Nigeria which include sample from spiritual healers, spiritual treatments included: use of water (66.7%), biblical verses (66.7%), fasting and prayer (96.7%), counseling (90%), beating (40%), and occupational therapy (13.3%) [11]. Several studies have also found evidence supporting the claim that Traditional healers can deliver effective psychosocial therapies, enabling social engagement and enhancing coping mechanisms [9]. They concluded that these therapies may assist to relieve discomfort and improve minor symptoms of depression and anxiety, but that there is little evidence that they can alter the course of serious mental illnesses including bipolar and psychotic disorders. Countries with similar health systems like Zimbabwe, Ethiopia and Kenya have utilized and showed the effectiveness of traditional healers in treating mental illness [16]. In Zimbabwe with similar health system to Nigeria, they have found out that traditional healers may provide simple explanations, which are often absent in clinical consultations, and could provide a more holistic/spiritual concept of mental illness, which is more acceptable to patients and consistent with local cultural values and beliefs [17].

Despite their popularity and acceptance in rural areas, traditional healers have shown a reluctance to collaborate with conventional psychiatric services because they do not believe in the efficacy of therapies given by psychiatrists for problems whose genesis is regarded to be spiritual [18]. Furthermore, where Traditional healers play a role in care pathways, delays in getting official mental health services have been reported and the duration of untreated psychosis is linked to worsened results [14]. In addition, there have been reports of human rights violations at healing centers (such as physical restraints, including the use of shackles and manacles, food restrictions, isolation, recitations from sacred texts, incantations, rituals, sacrificial offerings, and exorcism), which are still widely used among traditional and religious leaders in Nigeria to treat mental illnesses [19].

Due to concerns about harmful practices and an absence of evidence on safety and effectiveness of traditional mental health care, collaboration has mostly not happened and people in rural communities continue to struggle with appropriate management of mental health illness [20]. However, traditional healers are an intricate part of Nigeria's social cultural fabric, and are extensively patronized, and they should not be ignored when it comes to treatment of mental illness, instead they should be engaged constructively to promote better understanding of mental illnesses, diagnosis, and possible referral, while at the same time discouraging harmful practices.

## 3 Recommendations

The fact that traditional healers are important providers of mental health requires a holistic approach. Nigeria needs to work towards:

1. Improved cultural acceptability, accessibility, and affordability: There is a need for an improved cultural acceptability, accessibility, and affordability of psychiatric and mental health care services through the traditional healers. This can be done by integrating the modern skills and knowledge of psychiatric skills which will also help upscale referral services to other health care settings when possible.
2. Documentation: traditional healing has faced a major challenge of non-documentation of services provided for follow-up or further medical attention or history. Thus, there is a need for improvement of documentation of procedures, history and all medical services they offer to their clients.
3. Education, Policy and Guideline creation on mental health community practice: Mental education and training should be provided to traditional healers, and treatment modalities as combined; for instance, a culturally appropriate justification for someone's depression would be offered, followed by the required prescription for a psychotherapy or medication. This could be established by providing regular training programmes through the Department of traditional medicine, by volunteer community health workers that have received formal mental health training. To be effective, any strategy would necessitate adequate training and continuing education for traditional medicine practitioners, as well as re-education for conventional medical practitioners. Training would need to emphasize on the importance for collaboration, patient engagement and referral, and the necessity of mutual respect and trust, in addition to providing knowledge concerning the identification and treatment of mental diseases.
4. Harmful avoidance: Traditional healing processes for psychiatric and mental health have been regarded as safe but with no scientific basis. There is a need for more research on the principle of therapeutics - do no harm - which is important in rolling out the next plan in the country.
5. Policy and Budgeting: Policy focus to improve traditional medicine should also include substantial funding and focus on traditional healers providing psychiatric and mental health services. The focus shift from recognition to integration and policy implementation should be put in place to provide appropriate guidance and framework on their work and how to improve it. It is also essential to explore ways to ensure public resources for capacity building and other activities are made available through timely and effective budget execution.

## 4 Conclusion

Given the significant shortage of mental health human resources, we advocate for the integration of mental health training, interventions, and psychoeducation into primary care settings, as outlined in Nigeria's National Mental Health Service Delivery Policy to reduce the burden posed by traditional healers. Traditional and conventional medicine healthcare services should also be fused into collaborative health-care services through policies, so patients who seek mental health services at the communities from traditional healers can be referred to other conventional health centers for better treatment.

## Ethical approval

None.

## Sources of funding

None.

## Author contributions

Yusuf Hassan Wada and Omolayo Anjorin conceptualised, collect data, write manuscript and approved the final manuscript.

## Registration of research studies

1. Name of the registry:
2. Unique Identifying number or registration ID:
3. Hyperlink to your specific registration (must be publicly accessible and will be checked):

## Guarantor

Omolayo Anjorin and Yusuf Hassan Wada.

## Consent

None.

## Provenance and peer review

Not commissioned, externally peer reviewed.

## Declaration of competing interest

We declare no conflict of interest.
